# A prognostic signature based on adenosine metabolism related genes for ovarian cancer

**DOI:** 10.3389/fonc.2022.1003512

**Published:** 2022-11-28

**Authors:** Weifeng Liang, Chao Zhou, Jingshu Wang, Jing Zhao, Fang Liu, Guoqiang Wang, Chunwei Xu, Yuzi Zhang, Wenxian Wang, Shangli Cai, Yusheng Han, Lei Chang, Peihai Zhang

**Affiliations:** ^1^ Department of Gynecology, Qilu Hospital (Qingdao), Cheeloo College of Medicine, Shandong University, Qingdao, Shandong, China; ^2^ Department of Bioinformatics and Biostatistics, Shanghai Jiao Tong University, Shanghai, China; ^3^ Medical Department, Burning Rock Biotech, Guangzhou, China; ^4^ Institute of Basic Medicine and Cancer (IBMC), Chinese Academy of Sciences, Hangzhou, Zhejiang, China; ^5^ Department of Clinical Trial, The Cancer Hospital of the University of Chinese Academy of Sciences (Zhejiang Cancer Hospital), Hangzhou, Zhejiang, China; ^6^ Department of Obstetrics and Gynecology, The First Affiliated Hospital of Zhengzhou University, Zhengzhou, Henan, China

**Keywords:** adenosine metabolism, ovarian cancer, gene expression, prognostic analysis, signature

## Abstract

**Background:**

Ovarian cancer is one of the most common cause of cancer death in women due to its late diagnosis and susceptibility to drug resistance. Adenosine (ADO) signaling plays a key role in immune activity and tumor progression. In this study, we constructed a signature of ADO metabolism related genes expression in patients with ovarian cancer.

**Methods:**

A total of 372 ovarian cancer patients from TCGA was used as training set and 1,137 patients from six GEO datasets were as validation set. The gene expression and drug response inhibitory concentration values for ovarian cancer cell line from GDSC were used for drug sensitivity analysis. The non-negative matrix factorization algorithm and ssGSVA were used to construct the ADO score.

**Results:**

Patients with high ADO score had shorter overall survival (OS) than those with low ADO score in both training set (HR = 1.42, 95% CI, 1.06-1.88) and validation sets (pooled HR = 1.24, 95% CI = 1.02-1.51). In GSEA analysis, genes in ATP synthesis related pathways were enriched in the low ADO score group (adjusted P value = 0.02). Further, we observed that the high ADO score group had significantly higher levels of most cancer hallmark signatures (all adjusted P values < 0.01) and T cell dysfunction and exclusion signatures than the low ADO score group (all adjusted P values < 0.001). Patients with lower ADO score tended to be sensitive to common drugs including Olaparib and Paclitaxel (adjusted P values = 0.05 and 0.04, respectively).

**Conclusions:**

In conclusion, the established ADO signature could be used as a prognostic biomarker to stratify ovarian cancer patients and had the potential to guide the drug exploitation and personalized therapy selection.

## Introduction

Ovarian cancer is one of the most common and lethal cancers (1), which ranks the eighth leading cause of cancer-related female deaths worldwide, accounting for 3.4% of all cancer deaths in women (2). Due to the non-specific symptoms at early stage, about 70% of ovarian cancer patients were diagnosed at advanced stage (3). The standard treatment for ovarian cancer is primary surgery and sequential platinum-based chemotherapy (4). In addition, targeted therapies are remarkable options for patients including PARP inhibitors targeting BRCA germline mutations, VEGF inhibitors targeting tumor angiogenesis (5). However, because of late diagnosis and susceptibility to drug resistance, the recurrence rate is still high and the 5-year survival rate is only 49% (6). Therefore, it is necessary to identify the patients who have a higher probability to relapse and require advanced cancer intervention and managements.

Previous studies have demonstrated that adenosine (ADO) signaling has immunosuppressive effects by suppressing the activity of natural killer cells (NK) and CD8+ cells and enhancing the polarization of dendritic cells and the proliferation of myeloid-derived suppressor cells (MDSC) (7, 8). In ovarian cancers, CD39 and CD73, responsible for adenosine production are over-expressed in tumor tissues and associated with worse prognosis (9). Besides, based on a murine model and patients cohort of ovarian cancer, it was found that CD73 expression on ovarian tumor cells and cancer associated fibroblast cells (CAFs) promoted cancer cell survival and immune escape (10). Further, accumulated extracellular ADO induces immunosuppressive effects, mainly through interacting with four G protein-coupled receptors: ADORA1, ADORA2A, ADORA2B, and ADORA3 (11). For example, ADORA2B activation in MDSCs significantly induces VEGF secretion and angiogenesis (12). In addition, ADORA1 antagonist tended to reduce the cisplatin resistance in ovarian cell lines (13). Therefore, ADO metabolism played an important role in ovarian cancer. In consideration that ADO metabolism is not only regulated by different adenosine-producing enzymes, but also by counteracting ATP-regenerating pathways, adenosine degrading pathways, and cellular adenosine uptake (8), it is rational to develop a signature with the integration of multiple adenosine metabolism related genes expression for the prognosis prediction. We hypothesized that the signature characterized as the conversion from ATP to ADO might be related to immunosuppressive state and worse prognosis.

In this study, we developed and validated an ADO metabolism related signature to predict prognosis in ovarian cancer based on the expression data of TCGA and six GEO datasets. Further, in order to explore the potential mechanism and clinical utility, we analyzed the association of ADO score with immune microenvironment and drug sensitivity.

## Materials and methods

### Data download

Twenty-two ADO metabolism related genes (18 ADO metabolism enzymes and 4 ADO receptor) were collected from a previous study (8) and the description of their biological function is shown in **Supplementary Table 1**. A total of 372 ovarian cancer patients from TCGA was used as training set and 1,137 patients from 6 GEO datasets were as validation set. The RNAseq, whole-exon sequencing (WES), and corresponding clinical data of TCGA were downloaded from portal.gdc.cancer.gov, and the six GEO validation datasets (GSE14764, GSE23554, GSE26712, GSE32062, GSE49997, GSE140082) were downloaded from www.ncbi.nlm.nih.gov/gds. The basic information of these cohorts is summarized in **Supplementary Table 2**. The gene expression and drug response inhibitory concentration (IC50) values for 198 drugs in ovarian cancer cell lines were obtained from Genomics of Drug Sensitivity in Cancer (GDSC, www.cancerrxgene.org) (14).

### Biomarker discovery and signature construction

We used the non-negative matrix factorization (NMF) algorithm to obtain adenosine related Meta genes based on the expression data from TCGA. In detail, the R package “NMF” (15) with the default methods ‘brunet’ was applied. The rank was set as 2 to 10 and the number of runs for each rank set was 200. The cophenetic correlation coefficient was used to determine optimal clustering number. The factorization rank was selected once cophenetic correlation coefficient started to decrease (16). After that, we extracted the top 15 genes for each basis from the results of NMF which were used for the downstream analysis. A total of 20 genes was included in the signature construction. Two gene clusters were identified by consensus clustering algorithm with a correlation matrix of genes expression (17). Further, we calculated the enrichment score by ssGSVA method implemented in R package ‘GSVA’ (18) with the above two gene clusters respectively and defined the ADO score as the difference between the enrichment score of two gene clusters.

### Prognostic analysis

Patients were classified into high and low ADO groups by the median of ADO score in each cohort. Kaplan-Meier survival curve and univariable/multivariable Cox regression model were used to analyze the association between ADO score and overall survival (OS). In order to explore the interaction effects of BRCA1/BRCA2 mutation and Homologous Recombination Deficiency (HRD) with ADO score, hierarchical analysis was applied and interaction P value was evaluated by Wald test. The Number of Telomeric Allelic Imbalances (NtAI) count, Large-scale State Transitions (LST) count, Homologous Recombination Deficiency (HRD-LOH) score and HRD score for TCGA samples were obtained from Andrea M Marquards’ article (19). In the validation set, meta-analysis with random effect was used to pool the hazard ratio (HR) of the six GEO datasets.

### Molecular mechanisms and drug sensitivity analysis

To explore the molecular mechanism, the gene set enrichment analysis (GSEA) was performed in TCGA patients to analyze the significantly enriched pathways in high and low ADO score groups. In addition, the association between ADO score and ten cancer hallmark signatures related Gene Ontology (GO) terms were also analyzed (20). In order to explore the relationship between ADO score and immune microenvironment, immune infiltration signature calculated by CIBERSORT (21), ESTIMATE (22), MCPcounter (23), single-sample gene set enrichment analysis (ssGSEA) (24), TIMER (25) and TIDE (26) were compared between two ADO groups using Wilcoxon rank-sum test. The gene expression of immune checkpoint was similarly analyzed. For drug sensitivity, drugs with missing value in more than 80% of the samples were removed from the analysis. Finally, 180 drugs involving 23 pathways were used for Pearson correlation analysis between ADO score and IC50. Benjamini-Hochberg was used for P value correction for multiple comparisons.

### Statistical analysis

**Figure 1:**
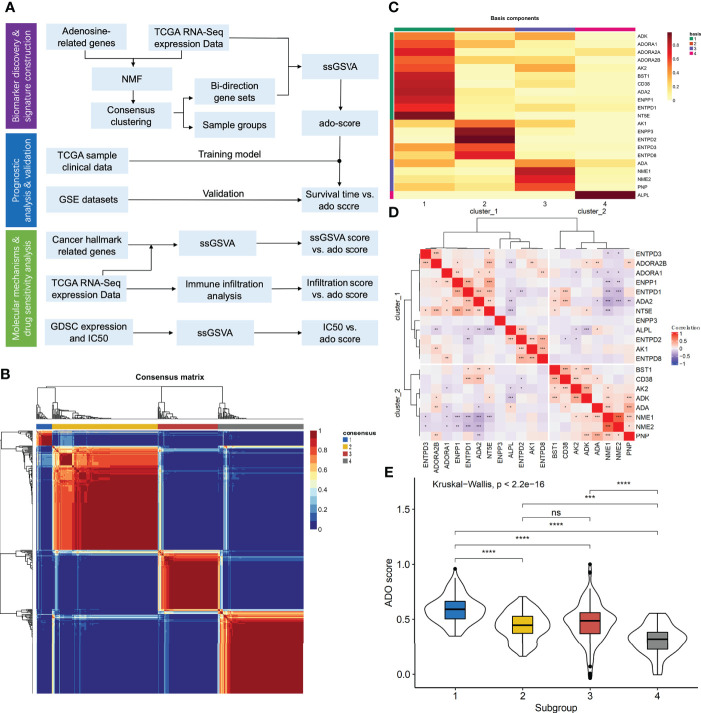
The construction of adenosine signature. **(A)** The flowchart of the study; **(B)** The consensus map of NMF clustering; **(C)** The metagenes obtained from NMF cluster; **(D)** The correlation matrix of adenosine metabolism related genes expression; **(E)** The boxplots of ADO score for different samples clusters. (ns: P > 0.05, *P < 0.05, **P < 0.01, ***P < 0.001, ****P < 0.0001).

The flowchart of this study is demonstrated in **Figure 1A**. All analyses were performed with R version 4.0.3. The Wilcoxon rank-sum test was used to test difference for all the calculated score or the gene expression between two groups if not specifically described. The P value less than 0.05 was regard as statistically significant and all the tests were two-side.

## Results

### Construction of the prognostic signature in TCGA dataset

For the 22 ADO related genes, only 38 of 434 (8.76%) ovarian cancer samples had non-silent mutation in these genes (**Supplementary Figure 1A**). Among them, mutation frequency of ENPP1 was the highest, which occurred in 5 of 434 samples (1.46%). There was no mutation event in ADA2, ENTP2, ENTPD8, and NME1 (**Supplementary Figure 1A**). For the copy number variants, it showed that the copy number variants were ubiquitous for ADO genes which indicated the mRNA expression of ADO genes might be discrepant among samples (**Supplementary Figure 1B**). According to the expression data of ADO genes, patients could be classified into four clusters in consensus cluster analysis (**Figure 1B**). Through NMF analysis, the optimal cluster number was set as 4 according to cophenetic correlation coefficient (**Supplementary Figure 2A**). The four Metagenes of ADO genes had different expression patterns and showed discrepant expression in all samples (**Figure 1C**, **Supplementary Figure 2B**). Top 15 genes in each metagene were extracted and formed a gene list for the following analysis. According to the correlation among these genes, we got two ADO gene groups (**Figure 1D**). In gene set 1, 8 of 12 genes were involved in the generation of ADP, AMP or ADO. However, in gene set 2, expect for ADA gene, the other 7 genes were enriched in the pathways related to ADP generation or ADO degradation (**Supplementary Table 1**). Furthermore, the enrichment scores by ssGSVA were calculated for the two gene sets, respectively. The ADO score was defined as enrichment score of gene set 1 minus gene set 2, which was significantly different among the four sample clusters (**Figure 1E**).

### Prognostic analysis in TCGA and GEO datasets

**Figure 2:**
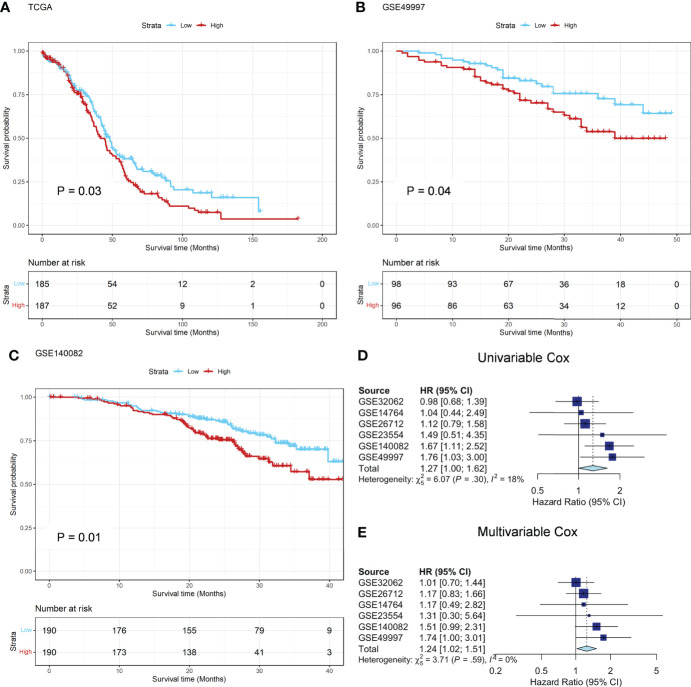
Prognostic analysis in TCGA and GEO datasets. **(A)** The Kaplan-Meier survival curves of OS between the ADO groups in the TCGA training dataset; **(B, C)** The Kaplan-Meier survival curves of OS between the two ADO groups in the GSE49997 and GSE140082 validation datasets, respectively; **(D, E)** The forest plot of the meta-analysis on the results of 6 GEO validation dataset calculated by using univariate and multivariate Cox model, respectively.

In TCGA cohort, patients with high ADO score had shorter OS than those with low ADO score (HR, 1.33; 95% CI, 1.02-1.72, **Figure 2A**). After adjusted for age, grade, stage, BRCA1/2 mutation and debulking surgery, the association was still significant (HR, 1.42; 95% CI, 1.06-1.88, **Supplementary Table 3**). Among the six validation cohorts, similar results were observed in GSE49997 and GSE140082 dataset (Log-rank P = 0.04 for GSE49997 and 0.01 for GSE140082, **Figures 2B** **, C**). In the meta-analysis of the six validation cohorts, the pooled HR was still significant in both univariable (HR, 1.27; 95% CI, 1.00-1.62; **Figure 2D**) and multivariable Cox models adjusted for age, stage, debulking surgery, histology and histological grade (HR, 1.24; 95% CI, 1.02-1.51, **Figure 2E**). The detailed results were reported in **Supplementary Table 3**. Besides, the hierarchical meta-analysis was applied stratified by serous and non-serous cancers, different grade and different stage, and the high ADO score was still significantly associated with worse OS in subgroups of stage III/IV (HR, 1.24; 95% CI, 1.01-1.52; **Supplementary Table 4**) and serous histology (HR, 1.23; 95% CI, 1.00-1.50; **Supplementary Table 4**) in the multivariable model. In grade G3/4 subgroup, the association were similar and borderline significant (HR, 1.28; 95% CI, 0.96-1.70; **Supplementary Table 4**).

### Hierarchical analysis for BRCA1/2 mutation and HRD score in TCGA dataset

**Figure 3:**
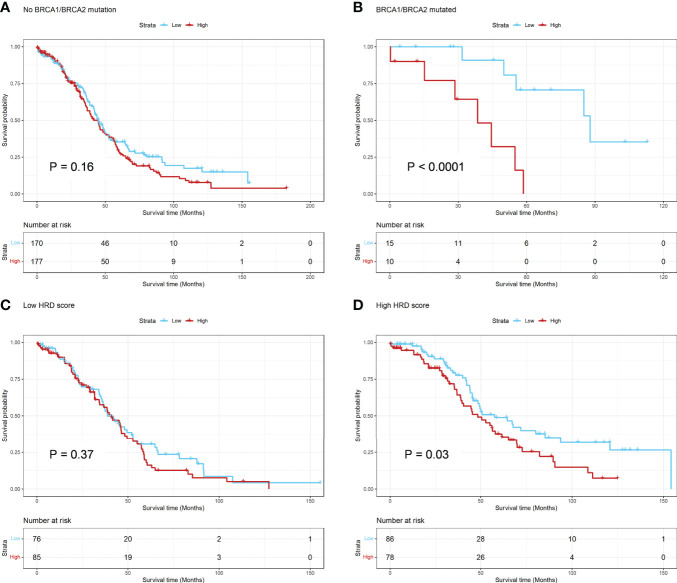
Hierarchical Analysis for BRCA1/2 mutation and HRD Score in TCGA dataset. **(A, B)** The Kaplan-Meier survival curve of OS between the ADO groups for subgroups of patients with or without BRCA1/2 mutation, respectively; **(C, D)** The Kaplan-Meier survival curve of OS between the ADO groups for subgroups of patients with low or high HRD score, respectively.

There is no significant difference in BRCA1/2 mutation frequency, NtAI count, LST count, HRD-LOH score and HRD between low and high ADO score groups (**Supplementary Figures 3A, B** **)**. For BRCA1/2 mutation, the association of ADO score and OS was significant in the patients with BRCA1/2 mutation, but not significant in other patients (P interaction = 0.018) (**Figures 3A** **, B**, **Supplementary Table 5**). Besides, the patients were classified in two subgroups based on the median of HRD score. There was a significant association between ADO score and OS in high HRD subgroup, but not in low HRD subgroup. However, the P interaction was not significant (P interaction = 0.35, **Figures 3C** **, D**, **Supplementary Table 5**). The results showed that ADO score tended to be significantly associated with prolonged survival only in BRCA1/2 mutant or HRD patients.

### The association between ADO score and biological signatures or immune genes expression in TCGA dataset

**Figure 4:**
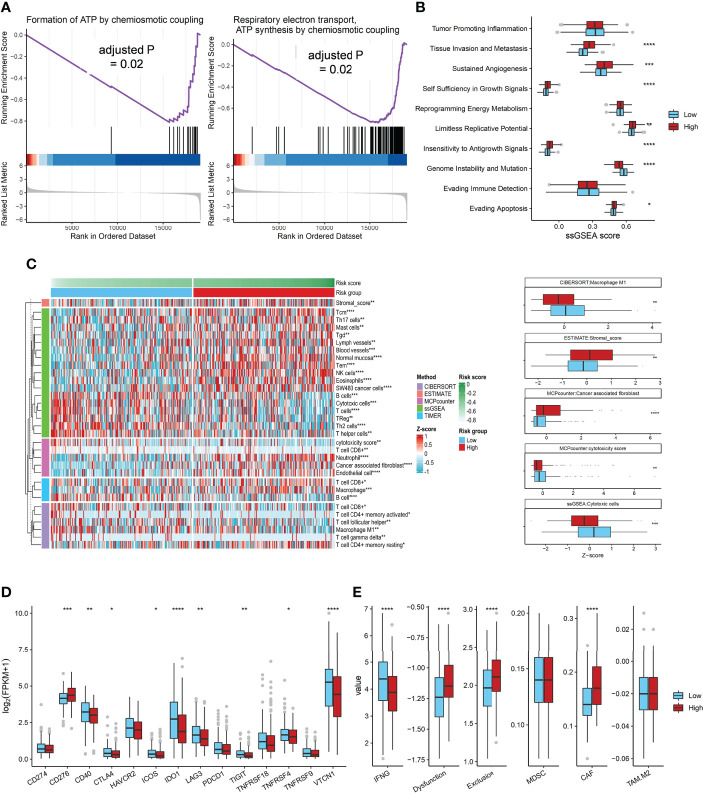
The Association of ADO score with biological signatures or immune genes expression in TCGA dataset. **(A)** Two significantly enriched ATP synthesis pathways in the low ADO group by GSEA; **(B)** Boxplots of the ssGSEA score of 10 cancer hallmarks signatures between two ADO groups; **(C)** Heatmap and boxplots of the infiltrated immune signatures score based on five algorithms; **(D)** Boxplots of immune checkpoint genes expression between two ADO groups; **(E)** Boxplots of the T cells dysfunction and exclusion signatures from TIDE algorithm between two ADO groups. (*P < 0.05, **P < 0.01, ***P < 0.001, ****P < 0.0001).

According to GSEA analysis, we found that genes in ATP synthesis related pathways were significantly enriched in the low ADO score group (adjusted P value = 0.02, **Figure 4A**). The associations of ADO score with ten cancer hallmarks signatures were analyzed (**Figure 4B**). Patients with high ADO scores had higher tissue invasion and metastasis, sustained angiogenesis, self-sufficiency of growth signals, but lower genome instability than those with low ADO scores (all adjusted P values < 0.01, **Figure 4B**, **Supplementary Table 6**). For ovarian cancer, patients with high genome instability tend to prolong OS (27, 28). Consistently, we found that patients with high HRD score had better prognosis (Log-rank P < 0.001, **Supplementary Figure 4**).

In order to explore the association between ADO scores and immune microenvironment, five immune signatures algorithms were applied. The heatmap showed that the high ADO group had enriched immunosuppressive cells, including stromal cells and CAF, while the low ADO group had enriched immune activating cells, including cytotoxic cells and M1 macrophage cells (adjusted P value = 0.01 for stromal cells, <0.001 for CAF, <0.001 for cytotoxic cells, 0.03 for M1 macrophage cells, **Figure 4C**, **Supplementary Figure 5**, **Supplementary Table 6**). Besides, for the immune checkpoint genes, compared with low ADO group, the high ADO group had higher expression of CD276, but lower expression of other genes, including CD40, IDO1, LAG3 and TIGIT (adjusted P value = 0.001, 0.02, <0.001, 0.05 and 0.02 respectively, **Figure 4D**, **Supplementary Table 6**). Further, based on the TIDE algorithm which could estimate the overall immune status, similar results were found. In detail, IFNG, representative of immune activating status was significantly higher in low ADO group than in high ADO group, while the signatures of T cell dysfunction, T cell exclusion and CAF were significantly lower in low ADO group (all adjusted P values < 0.001, **Figure 4E**, **Supplementary Table 6**).

### The association between ADO score and drug sensitivity in GDSC dataset

**Figure 5:**
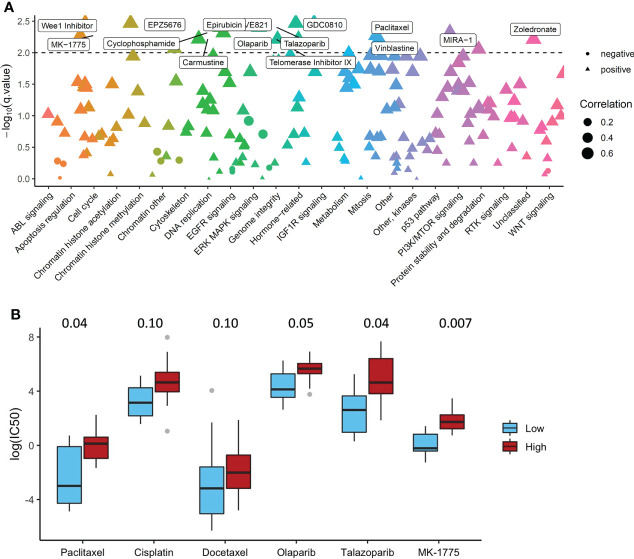
The Association of ADO score with Drug Sensitivity in GDSC dataset. **(A)** The Pearson correlation between ADO score and IC50 of the drugs in different biological pathways; **(B)** Boxplots of the IC50 for conventional and investigational drugs (P values were adjusted by Benjamini-Hochberg).

We constructed ADO score based on the expression data of DGSC ovarian cancer cell lines and calculated the Pearson correlation between ADO score and IC50 of 180 drugs. A total of 26 cell lines was used to obtain the IC50 (**Supplementary Table 7**). IC50 of 65 drugs was significantly associated with ADO score after Benjamini-Hochberg correction (adjusted P value < 0.05, **Figure 5A** **).** The detailed adjusted P values are shown in **Supplementary Table 8**. High ADO score group had significant higher IC50 than low ADO score group for conventional drugs, including Talazoparib, Paclitaxel and Olaparib (adjusted P value = 0.04, 0.04 and 0.05 respectively, **Figure 5B**), while not for Cisplatin and Docetaxel (adjusted P value = 0.10 and 0.10 respectively, **Figure 5B**). For MK-1775 (WEE1 Inhibitor) which was researched in a phase II clinical trials (29), high ADO score group also had significant higher IC50 than low ADO score group (adjusted P value = 0.007 and 0.05, **Figure 5B**).

## Discussion

In this study, an ADO metabolism related gene signature was constructed based on TCGA dataset to predict prognosis of ovarian cancer, and was validated in six GEO dataset. Moreover, compared with the low ADO score group, the high ADO score group with worse prognosis had lower genome instability, higher immunosuppressive signatures and tended to be insensitive to olaparide and paclitaxel.

Tumor growth requires a lot of energy which mainly comes from the degradation of ATP (30). In this process, a large amount of ADO was produced due to the mediation of CD73-CD39 axis. Hypoxia, chronic inflammation and nutrient deprivation are all catalysts for the generation of ADO (31–33) which can promote tumor cell proliferation, inflammatory response, neo-angiogenesis, tumor invasion and metastasis, and EMT transformation through interplay with the corresponding G protein-coupled receptors (34–36). In this study, we observed that the low ADO score group, rather than the high ADO score group was enriched for genes in ATP synthesis related pathways which suggested that the high ADO score group tended to accumulate more ADO. Further, based on the cancer hallmarks analysis, the high ADO score group had higher signatures in tissue invasion and metastasis pathway, sustained angiogenesis pathway and the insensitivity to antigrowth signals pathway than the low ADO score group. All evidence supported that patients with high ADO score would suffer a worse prognosis.

Subsequently, we noticed that the high ADO score group showed an immunosuppressive state. Previous literature has reported that tumor cells tended to accumulate eADO in the tumor core to create an immunosuppressive microenvironment (8, 37). Interestingly, in the high ADO score group, the expression level of immune effector molecule IFNG decreased significantly, while the level of NK cells, a main member of tumor killing cells, was relatively high. Molecular mechanism related studies showed that although the NK cells gathered at the tumor site, they were prevented from killing tumor cells by eADO and A2AR hindering the nutritional, promoting and cytolytic activities of NK cells, and finally inhibiting the production of IFNG (38, 39). Besides, the level of immune CAF signature was higher in the high ADO score group, which was consistent with the previous finding that CD73 was often highly expressed in the stroma of ovarian tumor tissue samples, probably CAFs (10). There have been a lot of clinical trials to develop anti-CD73 drugs to improve the effectiveness of anti-PD-1/PD-L1 immune therapy (40). Furthermore, we also observed that the high ADO score group had higher PD-L1 gene expression and might be insensitive to most conventional drugs, including chemotherapy, PARP inhibitor and other targeted drugs. Therefore, for patients with high ADO scores, the combination of anti-CD73 and anti-PD-1/PD-L1 immune therapy might be a potential treatment selection. Definitely, more molecular studies and clinical trials are needed to verify the hypothesis.

In the hierarchical analysis, it showed that ADO score tended to be significantly associated with prolonged survival only in BRCA1/2 mutant or HRD patients. As is well-known, HRD including BRCA1/2 mutation is one biomarker to measure the genomic instability (41), a hallmark of human cancer. It has been demonstrated that the ADO pathway was related to genomic instability. ENPP1, a gene with the function of hydrolyzing the extracellular cGAMP was increasingly expressed in cancer cells with genomic instability (42). Moreover, genomic errors contributed by HRD could lead to the formation of micronuclear envelopes which are highly rupture-prone and further produce cytosolic double-stranded DNA (dsDNA) (43). Then, cytosolic dsDNA is sensed by cGAS to be converted to the cyclic dinucleotide cGAMP, which could be catalyzed by ENPP1 and NT5E to form adenosine (42). Consistent with the results, in HRD subgroup, high ADO score group had higher gene expression of ENPP1 and NT5E expression and was relatively less enriched for genes in ATP synthesis, which leaded to more immunosuppressive adenosine accumulation and worse prognosis. However, the sample size in BRCA1/2 mutant or HRD patients was limited in this study and more evidence was needed to confirm this finding. Furthermore, we found that high score group seemed to be insensitive to PARP inhibitors and WEE1 inhibitors. The mechanism for both types of drugs is to inhibit DNA damages repair, increase genomic instability and trigger cell apoptosis (29, 44), which might increase cytosolic dsDNA production and promote the immunosuppressive adenosine formation in high ADO score group featured with ATP degradation. Therefore, for high ADO score group, it was a potential research topic to explore whether combination treatment could be a more appropriate choice.

In this study, we constructed an ADO metabolism related gene signature and explore its association with prognosis, immune signatures and drug sensitivity. However, there were still some limitations in our study. Firstly, this study was a retrospective analysis of public databases. However, our findings were validated in six independent cohorts to reduce false-positive results and further explained by the association with cancer hallmarks and immune signatures to confirm the rationality of the ADO signature. Secondly, the censor rate was high in the TCGA cohort, but it was not significantly different between ADO groups (High vs Low: 34.22% vs 42.7%, P = 0.10) which reduced the impact on the survival analysis. Thirdly, although the multivariable model involved all the available clinical features as confounders, there might be other unexpected confounders. Fourthly, the mRNA expression in our study was based on different platforms, such as RNAseq or microarray, so the evidence would be more solid if it was validated with a single method, especially RT-PCR or IHC. Fifthly, the drug sensitivity analysis was based on cell lines data. Therefore, the value of ADO score in the choice of treatment strategies for ovarian cancer remains need to be further explored in clinical trials. In the future, prospective studies with large sample sizes are required to confirm the clinical utility of ADO score.

In conclusion, we studied the relationship between the expression of ADO metabolism related genes and the prognosis of patients with ovarian cancer. ADO score was constructed to predict prognosis and had the potential to guide the treatment selection. More studies are needed to further confirm the clinical value of ADO score.

## Data availability statement

Publicly available datasets were analyzed in this study. This data can be found here: www.ncbi.nlm.nih.gov/gds portal.gdc.cancer.gov www.cancerrxgene.org.

## Author contributions

The authors confirm contribution to the paper as follows: study conception and design: LC, PZ and WL; data collection: CZ, JW, JZ, CX, YZ, WW and YH; analysis and interpretation of results: WL, CZ, JW, JZ, FL, GW, SC, LC and PZ; and draft manuscript preparation: all authors. All authors contributed to the article and approved the submitted version.

## Acknowledgments

We thank all the volunteer patients, staffs and researchers in TCGA, GEO and GDSC for their contribution to the research data.

## Conflict of interest

The authors declare that the research was conducted in the absence of any commercial or financial relationships that could be construed as a potential conflict of interest.

## Publisher’s note

All claims expressed in this article are solely those of the authors and do not necessarily represent those of their affiliated organizations, or those of the publisher, the editors and the reviewers. Any product that may be evaluated in this article, or claim that may be made by its manufacturer, is not guaranteed or endorsed by the publisher.

## Supplementary material

Supplementary Table 1 Annotation of 22 adenosine metabolism related genes.

Supplementary Table 2 Description of clinical information for seven cohorts.

Supplementary Table 3 The detailed results of survival analysis from the univariate and multivariate Cox regression model.

Supplementary Table 4 The hierarchical meta-analysis of clinical features in GEO datasets.

Supplementary Table 5 The detailed results of the Cox regression model for hierarchical analysis based on BRCA1/2 mutation and HRD score.

Supplementary Table 6 The detailed correlation of ADO score groups and gene signatures.

Supplementary Table 7 The cell line list.

Supplementary Table 8 The correlation between the ADO signature and IC50 for 180 drugs in ovarian cell lines.
