# Supplementary material for: A prognostic signature based on adenosine metabolism related genes for ovarian cancer
Source: Front Oncol. 2022 Nov 28;12:1003512. doi: 10.3389/fonc.2022.1003512 (PMC9742553; doi:10.3389/fonc.2022.1003512)
Supplement: Supplementary file 1 [file DataSheet_1.pdf]

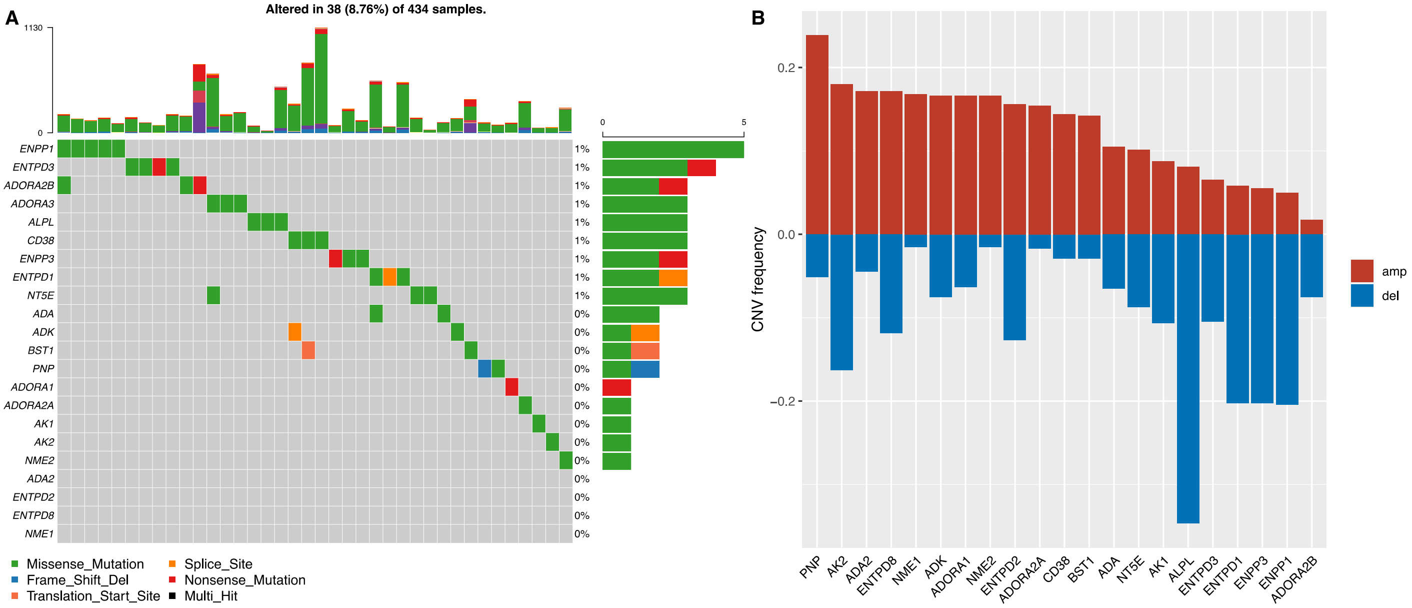

Supplementary Figure 1. The mutation and CNV frequency characteristic of the ovarian cancer samples in TCGA. (**A**) The waterfall plot for the 22 adenosine metabolism related genes; (**B**) The frequency of copy number amplification and deletion for these genes.

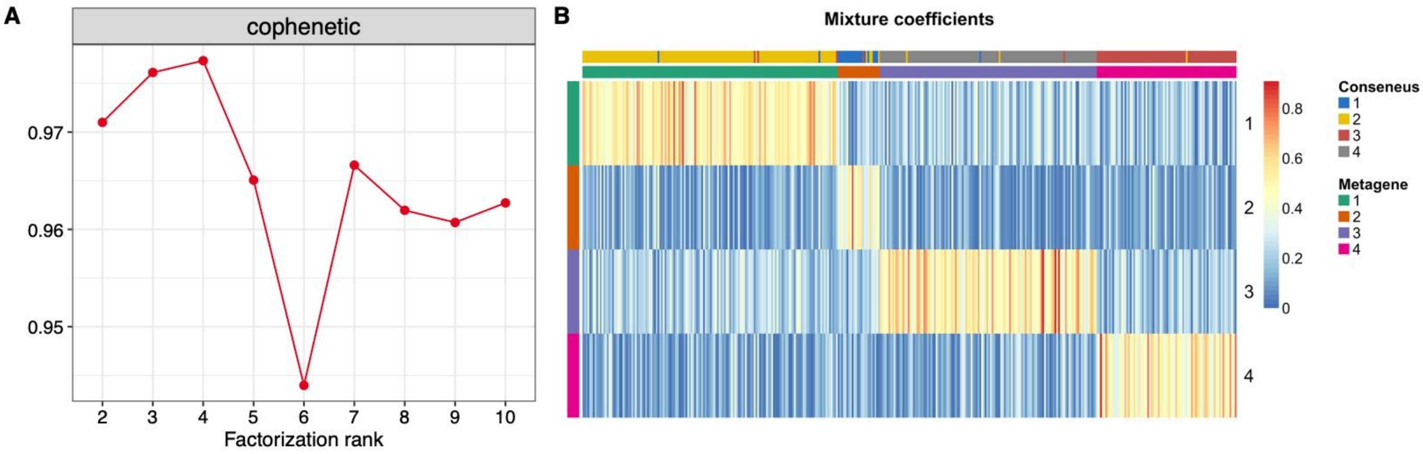

Supplementary Figure 2. Results of NMF cluster for TCGA ovarian cancer samples. (**A**) The cophenetic correlation coefficient at different factorization rank; (**B**) The heatmap of the Metagene expression for all samples.

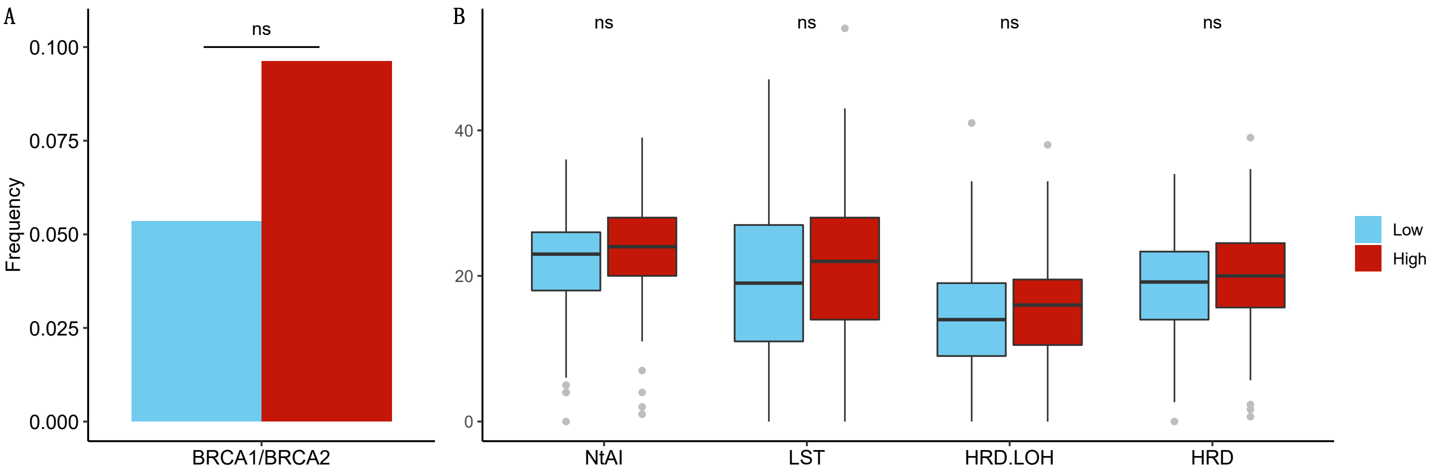

Supplementary Figure 3. Comparison of BRCA1/2 mutation frequency and genome instability between two ADO groups. (A) Barplot of BRCA1/2 mutation frequency; (B) Boxplots of Number of Telomeric Allelic Imbalances (NtAI) count, Large-scale State Transitions (LST) count, Homologous Recombination Deficiency (HRD-LOH) score, and the HRD score. (ns: $P > 0.05$, *$P < 0.05$, **$P < 0.01$, ***$P < 0.001$, ****$P < 0.0001$)

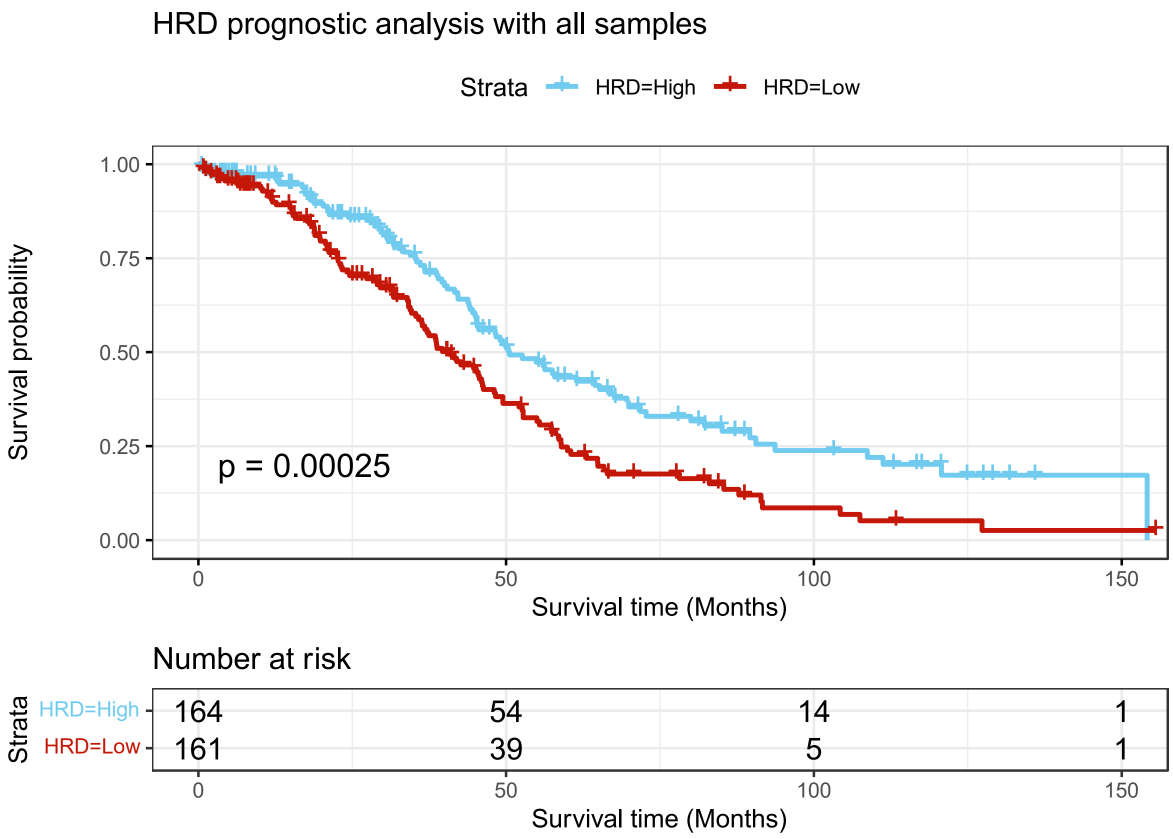

Supplementary Figure 4. Kaplan-Meier survival curve between HRD groups in TCGA dataset.

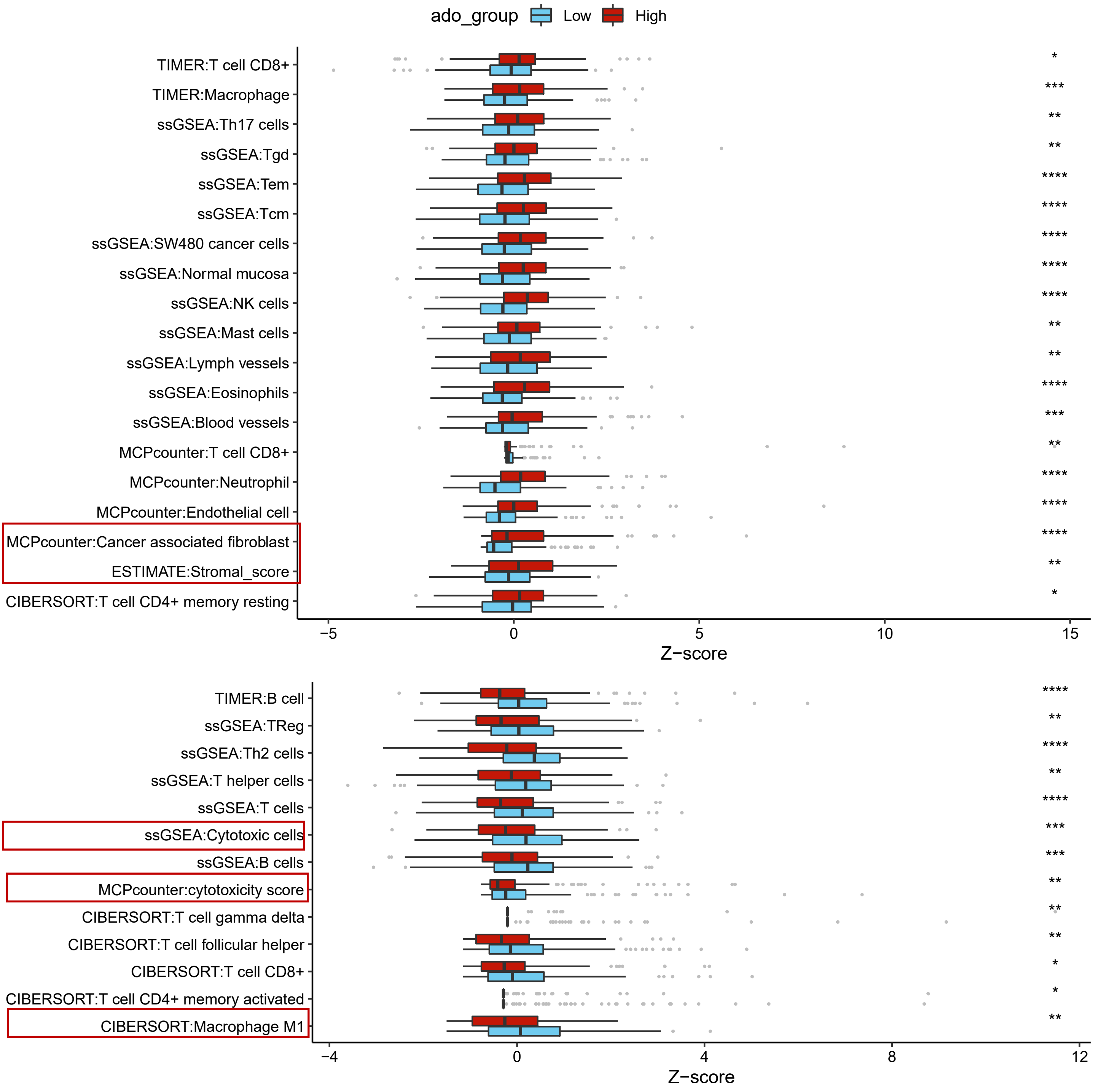

Supplementary Figure 5. Boxplots of ADO score and immune cell sigantures (*P < 0.05, **P < 0.01, ***P < 0.001, ****P < 0.0001)
